# Characterization of skin surface and dermal microbiota in dogs with mast cell tumor

**DOI:** 10.1038/s41598-020-69572-0

**Published:** 2020-07-28

**Authors:** Valentina Zamarian, Carlotta Catozzi, Anna Cuscó, Damiano Stefanello, Roberta Ferrari, Fabrizio Ceciliani, Olga Francino, Armand Sánchez, Valeria Grieco, Davide Zani, Andrea Talenti, Paola Crepaldi, Cristina Lecchi

**Affiliations:** 1 Dipartimento Di Medicina Veterinaria, Università Degli Studi Di Milano, Milan, Italy; 2 Vetgenomics. Ed Eureka. PRUAB. Campus UAB, Barcelona, Spain; 3 Molecular Genetics Veterinary Service (SVGM), Veterinary School, Universitat Autònoma de Barcelona, Barcelona, Spain; 4 The Roslin Institute, University of Edinburgh, Easter Bush Campus, Midlothian, EH25 9RG UK; 5 Department of Agricultural and Environment Science, Università Degli Studi Di Milano, Milan, Italy

**Keywords:** Skin cancer, Microbiome

## Abstract

The skin microbiota interacts with the host immune response to maintain the homeostasis. Changes in the skin microbiota are linked to the onset and the progression of several diseases, including tumors. We characterized the skin surface and dermal microbiota of 11 dogs affected by spontaneous mast cell tumor (MCT), using skin contralateral sites as intra-animal healthy controls. The microbial profile differed between healthy and tumor skin surfaces and dermis, demonstrating that the change in microbiota composition is related to the presence of MCT. The number of observed taxa between MCT and healthy skin surfaces was detected, showing a decrease in number and heterogeneity of taxa over the skin surface of MCT, at both inter- and intra-individual level. Preliminary data on bacterial population of MCT dermis, obtained only on three dogs, demonstrated an intra-individual reduction of taxa number when compared to the skin surface. Taxonomy reveals an increase of *Firmicutes* phylum and *Corynebacteriaceae* family in MCT skin surface when compared to the healthy contralateral. In conclusion, we demonstrate that microbial population of skin surface and dermis is related to mast cell tumor. Our study provides the basis for future investigations aiming to better define the interaction between mast cell tumors, microbiota and host immune response.

## Introduction

The epidermis, or skin surface, provides the external layer to the three parts of the skin, the inner layer being the dermis and hypodermis. The epidermis is regarded as a microenvironment containing a rich eukaryotic and prokaryotic population, currently defined as the microbiota^1,2^, which plays a role as protective and immunological barrier^3^.

Culture-independent determination of the microbial profile of dog skin surface has been recently determined, showing that microbiota largely differs between body sites^4^. The skin microbiota is modulated by extrinsic (e.g. diet, environment) and intrinsic (e.g. genetics) factors^5^, and is not only limited to skin surface but also extends to the dermis^6^. Remarkably, bacteria detected within the human healthy dermis and subcutaneous adipose tissue showed a different microbial population profile as compared to the skin surface^7^. Four major phyla—namely *Actinobacteria*, *Firmicutes*, *Proteobacteria*, *Bacteroidetes*—and four major families—namely *Corynebacteriaceae*, *Propionibacteriaceae*, *Staphylococcaceae*, *Micrococcaceae*—dominate both canine^2^ and human epidermal surfaces^1^.

Given the limited number of studies, the definition of canine skin “healthy microbiota” is still debated. Changes in microbiota composition are associated with the development of skin disorders in both humans and dogs^8,9^, as previously reported dermatitis^10^ in dogs.

The defensive capability of skin is supported by healthy and balanced microbiota through the interaction with the residing immune cells in the cutis^9,11,12^. Due to their localization within the skin, mast cells are in close contact with the bacterial population^13^. The activities of mast cells may be amplified when changes in microbiota composition occur^14^. The relationship between mast cells and skin surface microbiota^15^ has been poorly investigated, so far.

Canine Mast Cell Tumor (MCT) arises from an uncontrolled proliferation of neoplastic mast cells in cutaneous and subcutaneous tissues and is one of the most common skin neoplasms in dogs, accounting for up to 21% of all skin tumours^16^. The alterations of the microbiota of epidermal surface and its relationship with tumors have been investigated in humans and in experimental animals^17,18^. The onset of MCT and the presence of a high number of mast cells, the degranulation of which triggers a release of histamine, heparin and proteases, collectively defined as Darier’s signs^19^, could lead to changes in microbiota at both skin surface and dermis level. This hypothesis is supported by a study performed on P815, a mouse mastocytoma tumor model, showing that butyrate, an intestinal microbial metabolite, was able to modulate mast cells^20^.

To the best of our knowledge, no investigation has been carried out to characterize the skin surface microbiota of dogs affected by MCT. The presence of bacteria in the dermis of dogs remains unexplored as well. The present investigation aimed to profile the skin surface and dermis microbiota of owned-client dogs affected by spontaneous mast cell tumors in order to elucidate if and how the microbial community can change as associated with the neoplasm.

## Results

### Sequencing results

After sequencing, a total of 17 samples (8 skin swabs, 6 tumor dermal biopsies and 3 healthy tissue biopsies) showed a low reads count that led to their exclusion from subsequent analyses. The sequencing data of the remaining 19 samples from 9 dogs, including 14 skin swabs and 5 tumor dermal biopsies, produced a total of 1,906,333 reads and 5,869 features were obtained with an average of 54,399 (a minimum of 4,542 and a maximum of 459,808 sequences) after filtering. Detailed sequencing data for each sample are available in Supplementary Table S1 online.

### Skin surface microbiota

**Figure 1:**
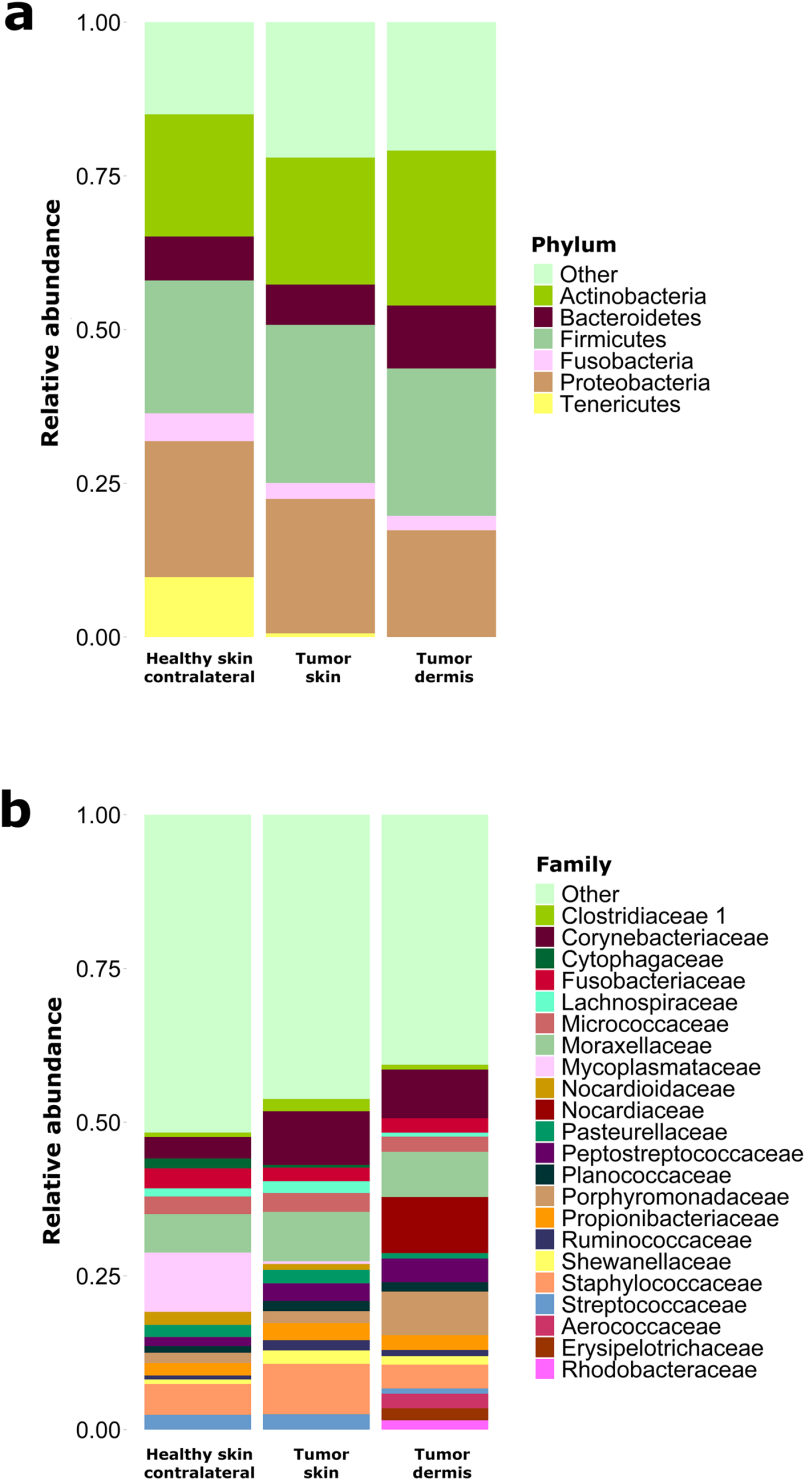
Skin surface and dermis taxonomy profile of MCT affected dogs. Taxonomy of seven healthy skin surfaces and seven tumor skin surfaces and five dermis is shown at phylum (**a**) and family (**b**) level, respectively.

Taxonomy results at phylum and family level are shown in Fig. 1a,b, respectively. An abundance of 2% was arbitrarily selected as the cut-off for the analysis. All taxa found at phylum and family level are provided in Supplementary Table S2 online. At the phylum level, the most representative taxa on dog skin surface were *Actinobacteria*, *Bacteroidetes*, *Firmicutes*, *Fusobacteria* and *Proteobacteria*. *Firmicutes* showed an increase in their abundance in MCT as compared to healthy skin samples (mean of 30% ± 4.8% and 21% ± 9.7% in tumor and healthy, respectively; *p* = 0.030). The most abundant families were *Corynebacteriaceae*, *Staphylococcaceae*, *Moraxellaceae*, *Mycoplasmataceae*. A statistically significant increase of *Corynebacteriaceae* was found on the tumor skin surface as compared to healthy contralateral (mean of 6.5% ± 3.4% and 2.4% ± 0.7% for tumor and healthy, respectively; *p* = 0.050).

The alpha diversity was investigated to study the species richness (number of OTU) and their relative abundance (Shannon index), whereas the beta diversity was calculated to assess the microbiota structure using qualitative (unweighted UniFrac) and quantitative (weighted UniFrac) approaches without taking into account the phylogeny (Bray–Curtis).

The expected mean percentage of observed taxa was 63.06% (confidence interval 59–67%) and 36.94% (confidence interval 33–40%) for healthy and tumor skin surface, respectively. Within alpha diversity analysis, the observed species decreased over the tumor skin surface (*p* < 0.001) at the Chi-square test (Supplementary Table S3 online). In 3 out of 5 samples, a significant reduction (*p* = 0.02) of observed taxa on the tumor skin surface respectively to the healthy skin contralateral was observed. Two animals showed a remarkably different pattern; dog D2 presented the same number of observed species in the MCT and the contralateral sample, whereas in dog D7 the number of observed species increased in MCT. Considering all samples, the mean and standard deviation of observed taxa among groups were 599 ± 324 and 351 ± 324 for healthy and tumor samples, respectively. Taking into account the microorganisms’ abundance, the groups did not show any variation in the Shannon index.

**Figure 2:**
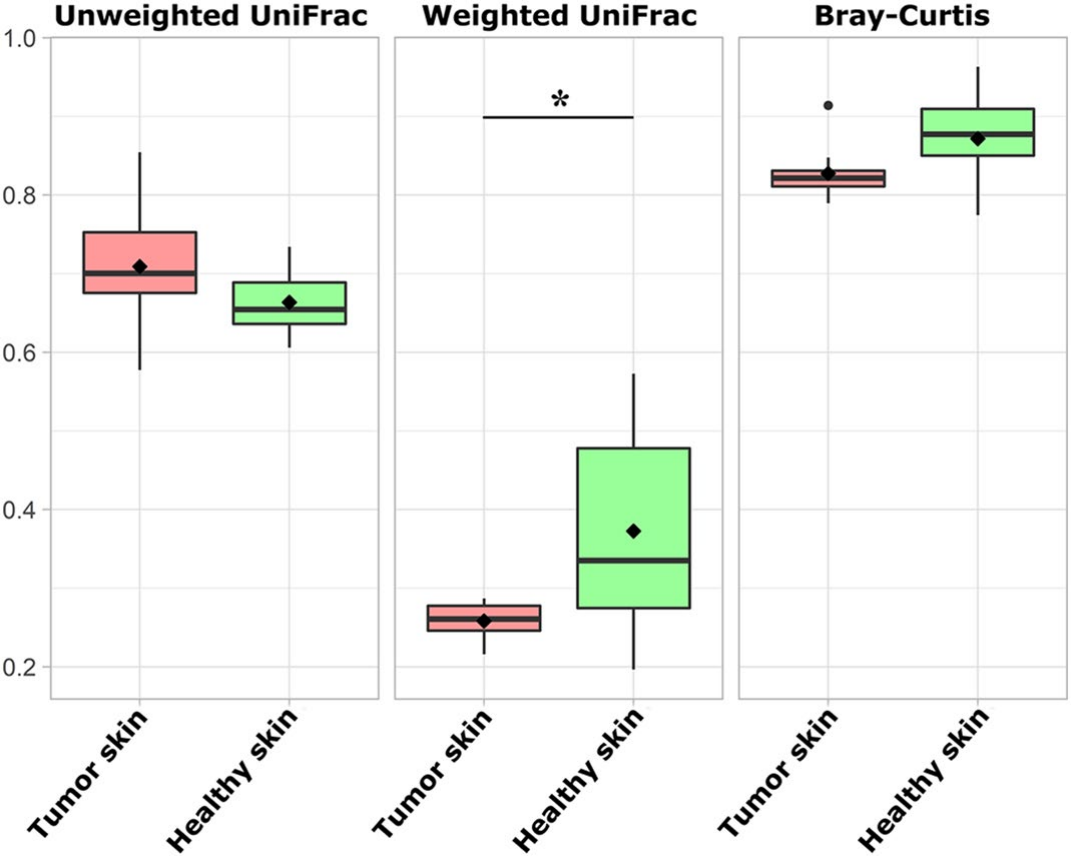
Box plots of unweighted, weighted UniFrac and Bray–Curtis distances between tumor skin and healthy skin surface groups. Weighted UniFrac matrix shows an intra-group variation in distances between healthy and tumor skin surface (*p* = 0.02).

Comparing the beta diversity of microbial structure in tumor and healthy skin surface, the dissimilarity was calculated by three distance matrices, namely unweighted UniFrac, weighted UniFrac and Bray–Curtis. Regarding the intra-group variation, a change in distances between healthy (mean of 0.37 ± 0.13) and tumor skin surface (mean of 0.26 ± 0.02) groups was observed in weighted UniFrac matrix (*p* = 0.02). Healthy skin samples present more divergent diversities than the more homogeneous diversities of MCT samples (Fig. 2).

Comparing the intra-animal distances, statistically significant changes in the microbiota structure were found for unweighted UniFrac (healthy vs tumor skin distance mean of 0.69 ± 0.08, *p* < 0.001), weighted UniFrac (healthy vs tumor skin distance mean of 0.29 ± 0.11, *p* = 0.004) and Bray–Curtis (healthy vs tumor skin distance mean of 0.78 ± 0.079, *p* < 0.001) distance methods (Supplementary Table S4 online).

**Figure 3:**
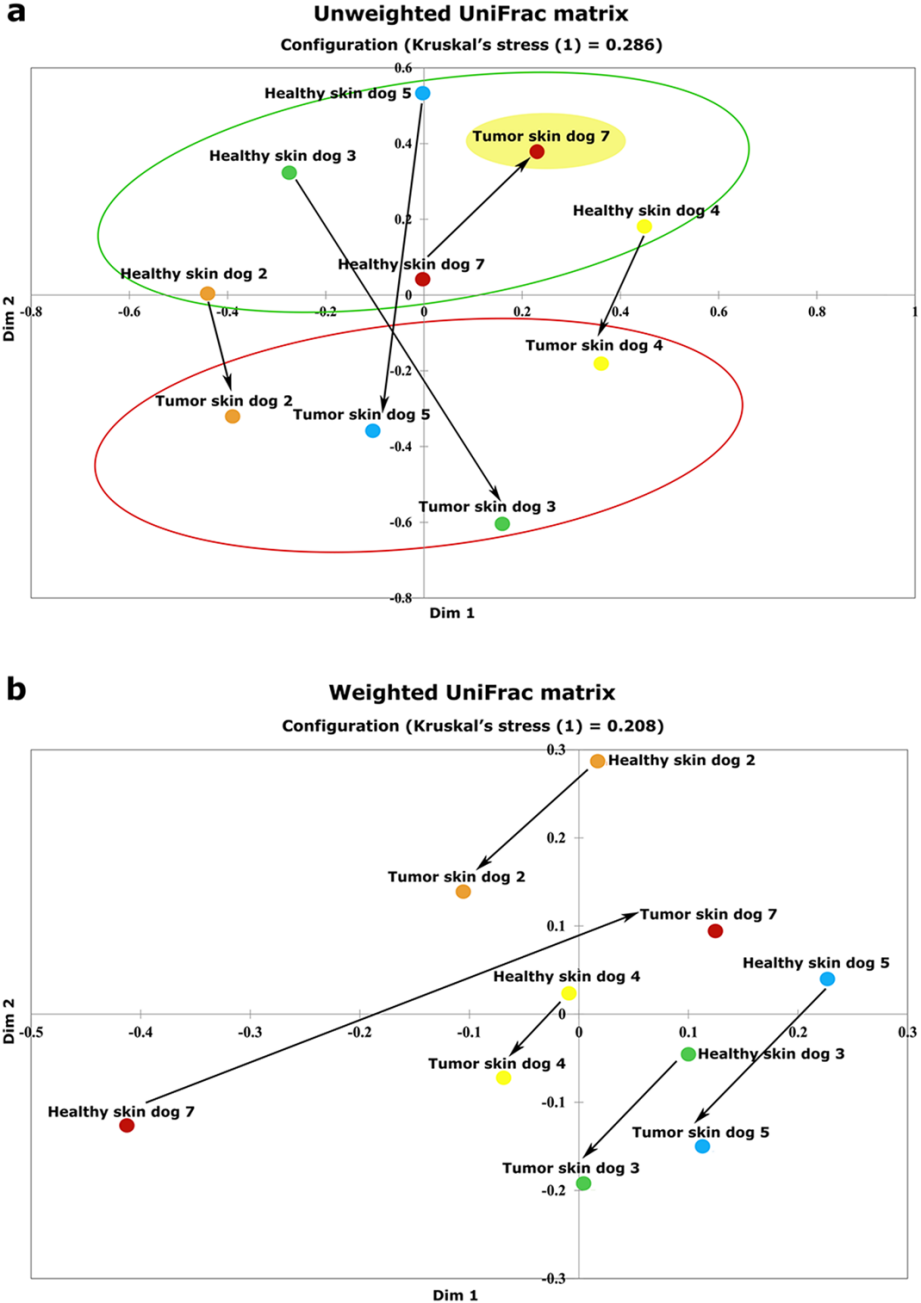
Multidimensional scaling plots of unweighted (**a**) and weighted (**b**) UniFrac distances comparing healthy (green circle) and tumor skin (red circle) surfaces. The arrows highlight the intra-animal shift from healthy and tumor skin surfaces.

The multi-dimensional scaling (MDS) analysis based on all distances present between skin samples of unweighted and weighted UniFrac distances is reported in Fig. 3a,b, respectively. A shift of the healthy and tumor skin surfaces paired samples towards the same direction in the second dimension of the plot in both matrices, highlighting a clear separation between tumor and healthy skin surface groups by the y-axis, is visible. As previously described in alpha diversity, dog D7 presented an opposite behavior also in beta diversity. MDS analysis was also shown using Bray–Curtis distances (Supplementary Figure S1 online).

**Figure 4:**
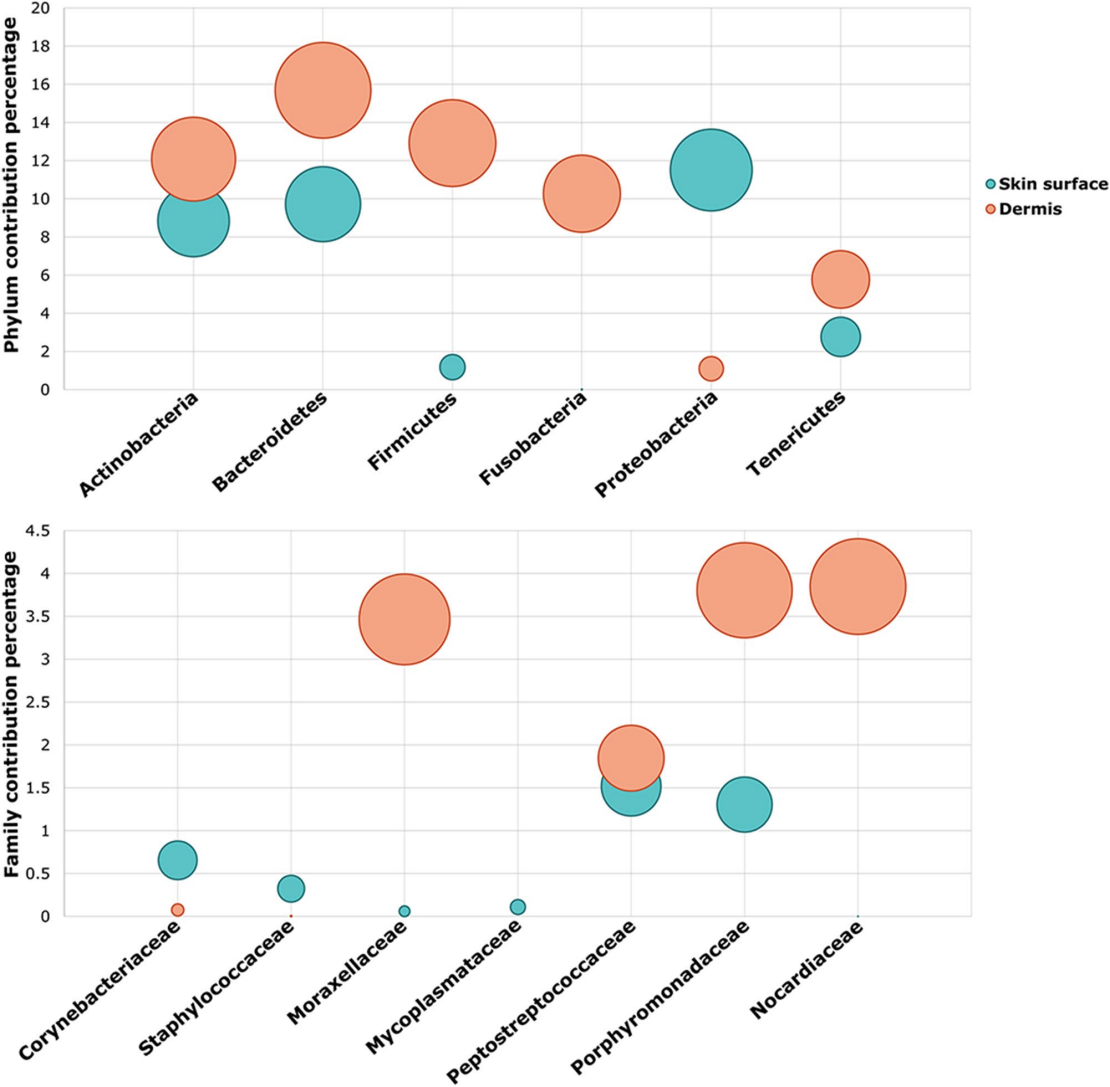
Balloon plots showing the contribution of the taxa (with a relative abundance ≥ 2%) which explain the shift from healthy to the tumor skin surface at phylum and family level.

The contribution of the taxa with a relative abundance ≥ 2% that mostly drove the shift from healthy to the tumor skin surface is presented in Fig. 4.

### Preliminary characterization of the microbiota composition of tumor dermis and comparison with its associated skin surface

After quality control step, the preliminary characterization of the microbiota composition of the tumor dermis was carried out on 5 tumor biopsies. An abundance of 2% was arbitrarily selected as the cut-off for the analysis. Results are presented in Fig. 1a,b for phylum and families, respectively. The most representative phyla of dermal microbiota were similar to those of skin surface. At family level, *Corynebacteriaceae*, *Staphylococcaceae*, *Moraxellaceae*,* Peptostreptococcaceae*, *Porphyromonadaceae* and *Nocardiaceae* were the most abundant.

Alpha and beta diversities between tumor dermis and the associated epidermal surface were also analyzed. A very preliminary statistical analysis was performed on alpha and beta diversity comparing the microbiota composition of the tumor dermis with overlying skin microbiota in 3 paired samples. Intra-animal chi-square test showed a difference in observed taxa between dermis and skin surface. The expected percentage of observed ASVs estimated by the test was 63.33% and 36.67% in skin surfaces and dermis, respectively (tumor skin swab CI: 58–67%; tumor dermis CI: 31–41%). A decrease in the number of ASVs detected in the dermis was found as compared to the corresponding skin surface (*p* < 0.001). The number of observed OTUs is presented in Supplementary Table S3 online. No difference was detected between dermis and skin surface microbiota using a quantitative test or analyzing the microbiota structure through the Shannon index. Intra-animal beta diversity analysis showed differences between tumor dermis and skin surfaces in unweighted (dermis vs tumor skin distance mean of 0.66 ± 0.09, *p* = 0.006), weighted (dermis vs tumor skin distance mean of 0.35 ± 0.14, *p* = 0.047) UniFrac and Bray–Curtis (dermis vs tumor skin distance mean of 0.80 ± 0.05, *p* = 0.001) matrices (Supplementary Table S4 online). No variation was found comparing dermis and skin intra-groups (data not shown). The contribution of the main taxa which drove the shift between tumor skin surface and dermis is shown in Fig. 4 and it is represented through the MDS plot of unweighted, weighted and the Bray–Curtis matrices, shown in Supplementary Figure S1 online.

### Core microbiota in skin surface and dermis of MCT affected dogs

**Figure 5:**
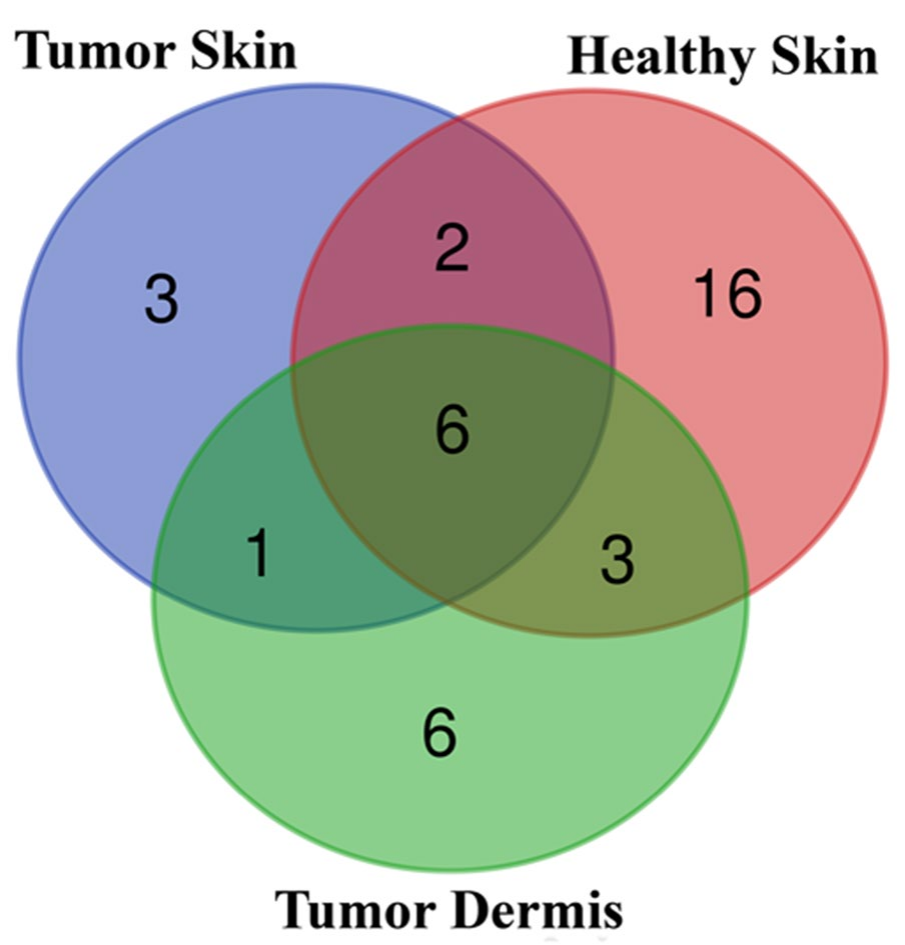
Venn diagram of ASVs, shared by tumor, healthy skin surface and tumor dermis which define core microbiota.

The core microbiota, defined as the bacterial taxa shared by all the analyzed samples, was identified in the tumor skin surface, contralateral healthy skin surfaces and in tumor dermis (Fig. 5). The core microbiota of tumor skin surface is composed of 12 ASVs, 6 of this shared with contralateral healthy skin surfaces and tumor dermis, belonged to 6 main families, namely *Corynebacteriaceae*, *Micrococcaceae*, *Propinibacteriaceae*, *Staphylococcaceae*, *Streptococaceae* and *Pseudomonadales*. Ten ASVs are present only in the tumor, 3 only on the tumor skin surface (*Moraxellaceae*, *Clostridiaceae*, *Pasteurellaceae*—families) and 6 only in the tumor dermis (*Nocardiaceae*, *Shewanellaceae*, *Enterobacteriaceae*, *Comamonadaceae*, *Corynebacteriaceae*—families). One ASVs is shared by tumor skin and dermis belonged to *Moraxallaceae* family.

## Discussion

In the present study, the microbial population over the skin surface and within the dermis of dogs affected by spontaneous mast cell tumor was investigated for the first time by determining the microbiota after amplification and sequencing of the V4 region of 16S rRNA^21^. The significant changes detected in the microbiota community provided the evidence that it is possible to discriminate between healthy and mast cell tumor site, even if with the limited number of samples included in this study.

Our results revealed that the number of observed OTUs decreased on the tumor skin surface compared to the healthy contralateral, suggesting a lost in the heterogeneity of microbial community on the skin surface over the tumor area.

Core microbiota of the healthy contralateral skin surface included more bacterial types (27 ASVs) than those of tumor skin surface or dermis (12 and 16 ASVs, respectively), confirming that a decrease in microbial diversity characterizes the tumor site, as already observed during an unhealthy status^22,23^.

We found that it is possible to discriminate the intra-patient tumor skin surface from healthy contralateral, as determined by beta diversity analysis using the weighted UniFrac matrix.

Several studies were carried out to investigate the relationship between microbiota and the development of skin disorders, including tumor and dermatitis. In the present study, an increase of *Firmicutes* phylum was observed, while investigation on human oral and pancreatic cancer^24^ reported a reduction of this phylum, highlighting that different cancers exert different selective pressures on the microbial population. Our results reported also an increase of *Corynebacteriaceae* family on the tumor skin surface compared to the healthy contralateral. Studies on dermatitis described a decrease in microbiota diversity^10^ with an increase of *Corynebacteriaceae*^25^, suggesting that a compromised skin barrier^26^ and local immune response^11^ are associated with changes in microbiota composition. All MCT samples included in this study featured an increase of *Corynebacteriaceae*, except dog 7, which had an opposite profile. Given the background that this was the only patient with ulcerations, we speculate that the development of a huge inflammatory reaction over the skin may potentially hamper the analyses and probably explain the inconsistent results, as previously described^27^.

Differences detected on the skin surface of MCT affected dogs suggest that tumor could interfere with the microbial population. The clonal proliferation of neoplastic mast cells^19,28^ and the presence of other immune cells^29^ infiltrating the tumor play a role in the host-microbiota interaction^30^. No data are available about the relationship between mast cells and surface microbiota. Skin microbiome can influence mast cell migration, localization and maturation in the skin^31^. A study on mouse skin microbiota supported its ability to recruit mast cells, promoting their maturation in dermis via stem cell factor production^32^. Furthermore, the activation of mast cells can be boosted by molecules produced by bacteria, such as δ-toxin released by *S.aureus* during atopic dermatitis^33,34^. The relationship between mast cells and microbiota is particularly interesting due to the microbiota capability to activate immune cells within the tumor microenvironment, and the potential exploiting of bacterial derived molecules to boost tumor immune defense^35^.

In the second part of the study, we focused on the presence of bacteria in dermis and on the comparison of dermis with the skin surface microbiota. The sequencing data demonstrated that bacteria were detectable in 5 out of 11 tumor dermal biopsies. On the contrary, the bacterial DNA was unquantifiable in the 3 healthy tissue biopsies, due to the low reads number. The limited number of case blocks any speculation about the possible relationship between dermis microbiota and the staging/grading of the MCT cases included in the study. In general, the presence of microbiota in the dermis has been poorly investigated so far, but having detected bacterial DNA in dermal compartment is a relevant finding for future investigation. Bacterial DNA was found in the dermal compartment^6^ and more recently human’s sebaceous and sweat glands were found to be inhabited by *Propionibacterium spp* and *Corynebacterium spp*, respectively^36^.

The comparison of the microbial population of tumor dermis and of tumor skin surface revealed a decrease in the number of OTUs in tumor dermis as well as a difference in intra-patient beta-diversity. Both these findings are corroborated by previously results, which reported that the skin surface is characterized by a greater bacterial abundance and community richness than biopsies^7^.

## Conclusions

The relationship between skin microbiota and MCT in dogs has yet to be fully elucidated. The next-generation sequencing approach allowed to demonstrate for the first time that the presence of MCT promotes an alteration of the epidermis microbiota structure and composition when compared to healthy skin; few differences were detected between tumor dermis and the associated epidermal surface microbiota, beside the decrease of ASV. Although the number of clinical case is limited, and the different stages and grades were not included in the statistical analysis, these preliminary findings pave the way to elucidate the relationship between mast cell tumor and composition of skin microbiota. Further studies on a larger number of patients are needed to support the reported results, which, if confirmed, have the potential to increase the knowledge of MCT pathophysiology and diagnostic.

## Materials and methods

### Ethics statement

The samples were collected at the Veterinary Teaching Hospital of the Università degli Studi di Milano from client-owned dogs that underwent veterinary consultation and surgery during the routine oncological management of canine mast cell tumor. All experimental procedures were reviewed and approved by the Ethics Committee of the University of Milano (approval number 118/19). Patients were recruited after written owner consent. All experiments were performed in accordance with the relevant guidelines and regulations.

### Studied population and sample collection

The experimental group was composed of 14 client-owned dogs, of which 11 were diagnosed with spontaneous MCT and three were healthy animals. All patients were companion dogs heterogeneous for the breed, age and gender. Detailed information about the enrolled animals is provided in Supplementary Table S5 online. Dogs with MCT were staged^37^ in order to exclude metastasis^38^ and were admitted to MCT wide margins excision and surgical removal of the sentinel lymph node. Tumors were histologically classified^39^ and grading was assessed^40,41^. In addition, the neoplastic involvement of sentinel lymph-node was assessed as previously described^42^.

From each MCT affected the dog, skin microbiota was collected using Sterile Dryswab (Medical wire) from the hairless area of the tumor and healthy contralateral site in the surgery room as previously described^4^. The surgical field was then scrubbed with an antiseptic solution of 2% chlorhexidine acetate as standard surgical preparation. To determine the dermis microbiota content, 4 to 5-mm punch biopsies from the center of the MCT mass, corresponding to dermis, were collected after the tumor excision and preserved in RNAlater solution (Sigma). Biopsies of cutaneous tissue derived from the margin of the surgical incision were collected from 3 healthy dogs that underwent elective sterilization, as healthy controls. Dermal biopsies from contralateral sites from MCT affected animals were not carried out for ethical reasons. Skin swabs and biopsies were then stored at − 80 °C until DNA extraction.

### DNA extraction, library preparation and sequencing

The DNA was extracted from skin swabs and tissue biopsies using DNeasy PowerSoil Kit (Qiagen, catalogue number 12888-100) following the manufacture’s general procedures, with some differences. Briefly, the skin swabs were cut directly into the PowerBead Tubes with Solution C1, vortexed and centrifuged at 10,000×*g* for 30 s; the supernatant was used for extraction. Twenty mg of the dermal biopsy were cut, treated with 20 µl of Proteinase K (Qiagen) and 60 µl of Buffer C1, incubated at 65 °C for 30 min and then transferred in the PowerBead Tubes to continue the procedure. Extraction blanks, processed like the other samples, were included for each extraction batch, as a control for contaminants. The DNA obtained from swabs and tissues were eluted in 30 µl and 60 µl of DNase-RNase free water, respectively, and stored at − 20 °C until use. DNA quantity and purity were checked using NanoDrop 1,000 Spectrophotometer (Thermo Scientific) at wavelengths 230, 260 and 280 nm.

V4 region of 16S rRNA gene was amplified using the following primer pair, the Forward primer—(5′-CCATCTCATCCCTGCGTGTCTCCGAC**TCAG**NNNNNNNNNNNNNNNNN**GAT**GTGYCAGCMGCCGCGGTAA-3′) composed of the adapter linker, the key, the sample-specific barcode and the F515 forward primer and the Reverse primer (5′-CTCTCTATGGGCAGTCGGTGATGGACTACNVGGGTWTCTAAT-3′) composed of the adapter linker and the R806 reverse primer. The Thermo Scientific Phusion Hot Start II High-Fidelity DNA polymerase kit (Thermo Fisher Scientific) was used to perform the PCR in 25 μl of the final volume. The mix contained RNAse and DNAse free water, 5 × Phusion Buffer HF (5 μl), dNTPs 2 mM (2.5 μl), Primer Fw 10 µM (1.25 μl), primer Rv 10 µM (1.25 μl) and Phusion High Fidelity Taq polymerase 2 U/μl (0.25 μl), and 5 ng of input DNA for dermal biopsy samples and 2.5 μl for the skin surface samples with an unquantifiable concentration. The thermal profile consisted of an initial denaturation of 30 s at 98 °C, followed by 27 cycles of 15 s at 98 °C, 15 s at 55 °C, 20 s at 72 °C and a final extension of 7 min at 72 °C. Quality and quantity of amplification were assessed in all samples, including extraction blanks and non-template controls (NTC), through Agilent Bioanalyzer 2,100 and Qubit fluorometer. The nucleic acid concentration of 12 (7 swabs and 5 biopsies) out of 33 samples was lower than 1 ng/μl after 27 cycles of PCR. Thus, an additional PCR was applied to these samples using the same condition increasing the number of cycles to 32. Each PCR reaction included an NTC to confirm the absence of contaminants in the reagents. The DNA sequencing was performed using an Ion Torrent Personal Genome Machine (PGM) with the Ion 318 Chip v2 (Thermo Fisher Scientific), following the manufacturer’s instructions. A mock community (ZymoBIOMICS Microbial Community DNA Standard, Zymo Research), as a positive control, was also sequenced to assess the quality of the run. In addition, 4 extraction blanks (2 from skin swabs DNA purification and 2 from tissue biopsy DNA purification) and 2 not template controls from the two PCR amplification reactions were sequenced to exclude the presence of contaminants in the reagents. Taxa considered as contaminants were excluded from all the samples analyzed (Supplementary methods).

### Quality control and sequence demultiplexing

Raw sequences were submitted to the National Center for Biotechnology Information under Bioproject accession number SUB6027391—Bioproject number: PRJNA555200. Raw data were imported into Quantitative Insight Into Microbial Ecology 2 software (QIIME 2; https://qiime2.org)^43^ for the analysis. Raw sequencing reads were demultiplexed to remove the Rv primer sequence and to associate each barcode to the correspondent sample. DADA2^44^ was used to denoise, dereplicate single-end sequences and remove chimaeras. Reads with a length of 253 bp were taken into account, considering the quality plot result and the V4 length mean of 250 bases. After that, the Amplicon Sequence Variants (ASVs), units of observation composed of unique sequences, were used to classify them and assign taxonomy using SILVA^45^ at 99% of Operational Taxonomic Units (OTUs) identity and trimmed to V4 region, as the reference database. The complete workflow is provided in Supplementary Methods online.

### Statistical analysis

Statistical analysis was performed using XLStat software for Windows (Addinsoft, New York, USA) on taxonomy and alpha and beta diversities. Data distribution was assessed using the Shapiro–Wilk test. From taxonomy results, the relative abundance of taxa present was determined in all samples. Alpha diversity describes the differences within samples or groups and indicates how many taxa are present by using a qualitative (observed species or observed ASVs) and a quantitative (Shannon index) approach. Beta diversity defines the differences between groups and takes into account how many taxa are shared between samples, generating distance matrices. We analyzed the beta diversity through unweighted and weighted UniFrac methods as a phylogenetic qualitative and quantitative approach, respectively and Bray Curtis analysis that does not consider the microbial community phylogeny. For both alpha and beta diversity, a sequence depth of 4,500 was applied.

The statistical analysis was performed on paired samples to assess the microbiota variation within the same individual. To characterize the core microbiota, we analyzed the shared ASVs between groups, considering the cutoff of 80% for the presence of the features within the groups. The difference between observed and expected features for healthy and tumor tissues was calculated by means of Chi-square test. T-test, Wilcoxon-signed- and Mann–Whitney-tests were applied based on the distribution of each dataset. Statistical significance was accepted at *p*-value ≤ 0.05.

For graphical representations, we plotted the shared ASVs using an online Venn tool (https://bioinformatics.psb.ugent.be/webtools/Venn/).

Multi-dimensional scaling (MDS) analysis was generated by means of XLStat software considering the distance matrix calculated on unweighted and weighted UniFrac and Bray–Curtis by QIIME2. The Kruskal’s stress (from 0 to 1) was taken into account to evaluate the goodness-of-fit of the test, with values closer to zero corresponding to a better and more reliable data representation. Box- and bar-plots were produced using R software (https://www.R-project.org) by the R package ggplot2 (https://cran.r-project.org/web/packages/ggplot2/, version 3.2.0) as shown in the Supplementary Methods online.

In addition to the previous multi-dimensional scaling, we produced a second MDS plot using the R cmdscale function on the Manhattan distances calculated on the relative abundance matrices for both samples and ASVs (both part of the R built-in stats package, R version 3.5.2). This step was carried out for both phyla and families separately, and for the skin and the dermal biopsy separately, for a total of four different analyses. Eigenvalues for the ASVs have been plotted as bar charts to identify the leading eigenvalues. Finally, a principal component analysis (PCA) using the PCA function in FactoMineR package^46^ (https://cran.r-project.org/web/packages/FactoMineR/, version 1.42), allowing for the detection of the percentage of contribution of every ASVs to the different components, was carried out. For this study, only the contribution to the first component was considered. The detailed bioinformatics workflow is available in Supplementary Methods online.

## Supplementary information

Supplementary file1 (PDF 720 kb)

Supplementary file2 (PDF 140 kb)

Supplementary file3 (XLSX 12 kb)

Supplementary file4 (XLSX 245 kb)

Supplementary file5 (PDF 552 kb)

Supplementary file6 (XLSX 17 kb)

Supplementary file7 (PDF 576 kb)
