# Supplementary material for: Characterization of skin surface and dermal microbiota in dogs with mast cell tumor
Source: Sci Rep. 2020 Jul 28;10:12634. doi: 10.1038/s41598-020-69572-0 (PMC7387470; doi:10.1038/s41598-020-69572-0)
Supplement: Supplementary file 1 — Supplementary file1 (PDF 720 kb) [file 41598_2020_69572_MOESM1_ESM.pdf]

Supplementary Figure S1.

Characterization of skin surface and dermal microbiota in dogs with mast cell tumor

Valentina Zamarian[1#], Carlotta Catozzi[1#], Anna Cuscó[2], Damiano Stefanello[1], Roberta Ferrari[1], Fabrizio Ceciliani[1], Olga Francino[3], Armand Sánchez[3], Valeria Grieco[1], Davide Zani[1], Andrea Talenti[4], Paola Crepaldi[5], Cristina Lecchi[1*]

(1) Dipartimento di Medicina Veterinaria, Università degli Studi di Milano, Milano, Italy

(2) Vetgenomics. Ed Eureka. PRUAB. Campus UAB, Barcelona, Spain

(3) Molecular Genetics Veterinary Service (SVGM), Veterinary School, Universitat Autònoma de Barcelona, Barcelona, Spain

(4) The Roslin Institute, University of Edinburgh, Easter Bush Campus, Midlothian, EH25 9RG, United Kingdom

(5) Department of Agricultural and Environment Science, Università degli Studi di Milano, Milano, Italy.

#Equal contribution

Supplementary Figure S1. Multidimensional scaling plots (a) of Bray-Curtis distances of the tumor and healthy skin surface and (b) of tumor skin surface and tumor dermis biopsy samples using Bray-Curtis matrix; (c) of unweighted and (d) weighted UniFrac distances comparing tumor dermis biopsy (orange circles) and the associated tumor skin surface (red cirles).

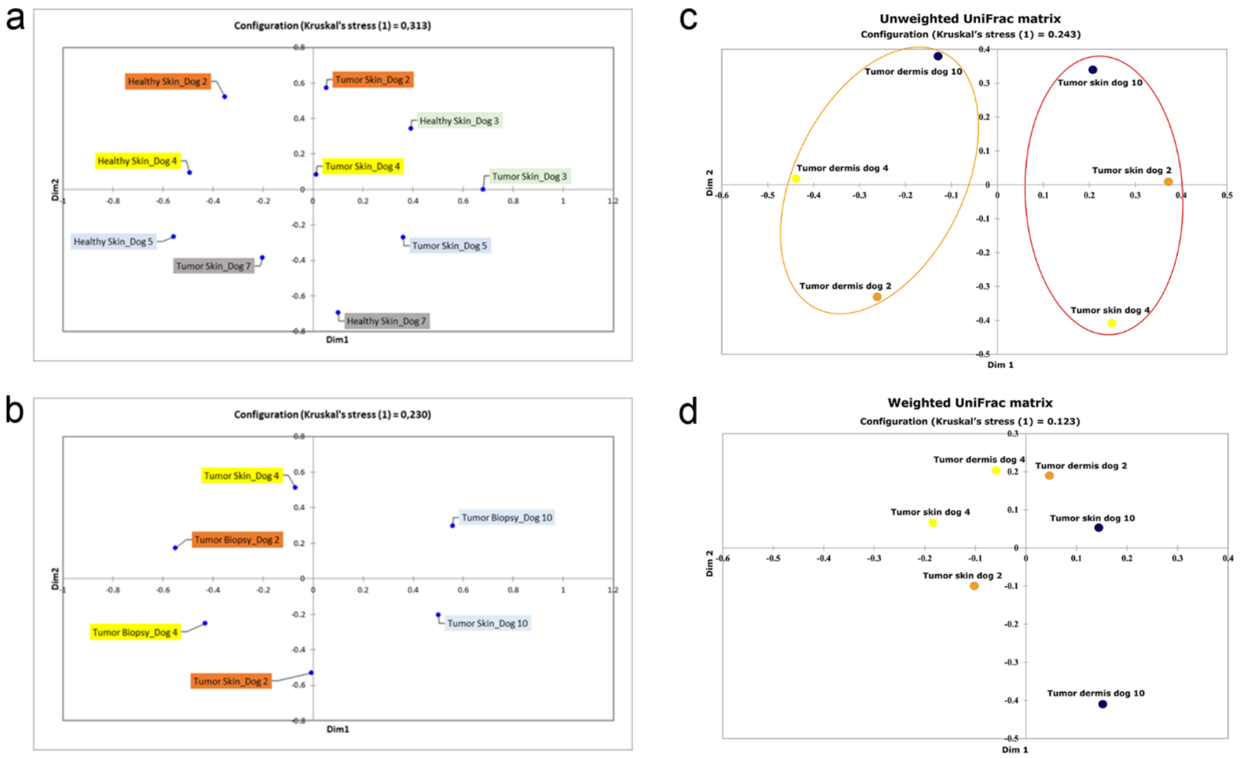
a
Configuration (Kruskal's stress (1) = 0,313)
Healthy Skin_Dog 2
Tumor Skin_Dog 2
Healthy Skin_Dog 3
Healthy Skin_Dog 4
Tumor Skin_Dog 4
Tumor Skin_Dog 3
Healthy Skin_Dog 5
Tumor Skin_Dog 5
Tumor Skin_Dog 7
Healthy Skin_Dog 7
Dim1
Dim2
b
Configuration (Kruskal's stress (1) = 0,230)
Tumor Skin_Dog 4
Tumor Biopsy_Dog 10
Tumor Biopsy_Dog 2
Tumor Biopsy_Dog 4
Tumor Skin_Dog 10
Tumor Skin_Dog 2
Dim1
Dim2
c
Unweighted UniFrac matrix
Configuration (Kruskal's stress (1) = 0.243)
Tumor dermis dog 10
Tumor skin dog 10
Tumor dermis dog 4
Tumor skin dog 2
Tumor dermis dog 2
Tumor skin dog 4
Dim 1
Dim 2
d
Weighted UniFrac matrix
Configuration (Kruskal's stress (1) = 0.123)
Tumor dermis dog 4
Tumor dermis dog 2
Tumor skin dog 4
Tumor skin dog 10
Tumor skin dog 2
Tumor dermis dog 10
Dim 1
Dim 2
