# Supplementary material for: Characterization of skin surface and dermal microbiota in dogs with mast cell tumor
Source: Sci Rep. 2020 Jul 28;10:12634. doi: 10.1038/s41598-020-69572-0 (PMC7387470; doi:10.1038/s41598-020-69572-0)
Supplement: Supplementary file 2 — Supplementary file2 (PDF 140 kb) [file 41598_2020_69572_MOESM2_ESM.pdf]

Bioinformatic Workflow - microbiota of dogs affected by Mast cell tumor

Considerations

Twelve samples out of 35 were not quantifiable after the V4-16S PCR. Therefore, the PCR was repeated for these samples - M003, M007, M009, M017, M025, M030, M031, M035, M043, M045 - with the same PCR conditions indicated in the manuscript but for 32 cycles. Even if extraction blanks and negative controls from the first PCR were unquantifiable, we decided to sequence them. We also sequenced the both negative controls from the two PCRs. Only the negative control from the second PCR (NTC2) was quantifiable after Qubit. After sequencing 19 samples were used to proceed in the analysis, taking into account a minimum read depth of 4500. We got a total number of sequences of 1,906,333 with a mean of 54,399 (minimum of 4,542 and a maximum of 459,808) and a number of features of 5,869. Sixteen samples were excluded due to the low number of reads, as well as the four extraction blanks and one PCR negative control.

QIIME2 workflow

QIIME2 webpage: https://qiime2.org

```
# Import Ion Torrent single-end fastq sequences
# Sequences file are in the directory 'casava-18-single-end-demultiplexed'
qiime tools import\
  --type 'SampleData[SequencesWithQuality]' \
  --input-path casava-18-single-end-demultiplexed \
  --input-format CasavaOneEightSingleLanePerSampleDirFmt \
  --output-path demux-single-endmtc.qza &

# Check the quality plot
qiime demux summarize \
  --i-data demux-single-endmtc.qza \
  --o-visualization demux-single-end_v2mtc.qzv &

# Denoise, dereplicate single-end sequences and remove chimeras
# Primer forward (trim-left) of 19 nucleotides
# Trunc-len value corresponds to the nucleotide truncation position
and depends on the quality plot and on the V4 mean length (about 250bp)
qiime dada2 denoise-single \
  --i-demultiplexed-seqs demux-single-endmtc.qza \
  --p-trim-left 19 \
  --p-trunc-len 253 \
  --p-n-threads 0 \
  --o-representative-sequences rep-seqs-V4mtc.qza \
  --o-table tableV4-dadamtc.qza \
  --o-denoising-stats mtcmicro &

# check the previous outputs
qiime feature-table tabulate-seqs \
  --i-data rep-seqs-V4mtc.qza \
  --o-visualization rep-seqs-V4mtc.qzv &

qiime feature-table summarize \
```

```
  --i-table tableV4-dadamtc.qza \
  --m-sample-metadata-file mtcmetadata.tsv \
  --o-visualization tableV4-dadamtc.qzv &

# SILVA database (Version 132) at 99% of Operational Taxonomic Units (OTUs)
identity and trimmed to V4 region
qiime tools import \
  --type 'FeatureData[Sequence]' \
  --input-path ./SILVA/99_otus_16S.fasta \
  --output-path ./SILVA/99_otus_16S.qza &

qiime tools import \
  --type 'FeatureData[Taxonomy]' \
  --input-format HeaderlessTSVTaxonomyFormat \
  --input-path ./SILVA/consensus_taxonomy_7_levels.txt \
  --output-path ./SILVA/ref-taxonomy99.qza &

qiime feature-classifier extract-reads \
  --i-sequences ./SILVA/99_otus_16S.qza \
  --p-f-primer GTGYCAGCMGCCGCGGTAA \
  --p-r-primer GGACTACNVGGGTWTCTAAT \
  --o-reads ./SILVA/refV4-seqs99.qza &

qiime feature-classifier fit-classifier-naive-bayes \
  --i-reference-reads ./SILVA/refV4-seqs99.qza \
  --i-reference-taxonomy ./SILVA/ref-taxonomy99.qza \
  --o-classifier ./SILVA/classifier_SILVA99_V4.qza &

qiime feature-classifier classify-sklearn \
  --i-classifier classifier_SILVA99_V4.qza \
  --i-reads rep-seqs-V4mtc.qza \
  --p-n-jobs -1 \
  --o-classification taxonomySILVA-dadaV4mtc.qza &

# Remove spaces from the file
qiime tools export \
  --input-path taxonomySILVA-dadaV4mtc.qza \
  --output-path taxonomy-with-spaces &

qiime metadata tabulate \
  --m-input-file taxonomy-with-spaces/taxonomy.tsv  \
  --o-visualization taxonomy-as-metadata.qzv &

qiime tools export \
  --input-path taxonomy-as-metadata.qzv \
  --output-path taxonomy-with-spaces &

qiime tools import \
  --type 'FeatureData[Taxonomy]' \
  --input-path ./taxonomy-with-spaces/metadata.tsv \
  --output-path newtaxonomySILVA-without-spacesmct.qza &

#visualize your output
qiime metadata tabulate \
```

```
  --m-input-file newtaxonomySILVA-without-spacesmct.qza \
  --o-visualization newtaxonomySILVA-without-spacesmct.qzv &

# Remove chloroplast sequences (Possible contaminats)
qiime taxa filter-table \
  --i-table tableV4-dadamtc.qza \
  --i-taxonomy newtaxonomySILVA-without-spacesmct.qza \
  --p-exclude Chloroplast \
  --o-filtered-table Taxonomy/tableV4-SILVA-noChlor.qza &

qiime feature-table summarize \
  --i-table ./Taxonomy/tableV4-SILVA-noChlor.qza \
  --o-visualization ./Taxonomy/tableV4-SILVA-noChlorMCT.qzv &
```

The NTC2 got a higher number of sequences as compared to the others. From the taxonomic profile, we took into account the samples run in the same plate and also the three wells really close to the negative control (M007 and M043, followed by M025). We noticed that Halomonadaceae and Flavomonadaceae taxa with a relative abundance of 19.3% and 8.7% respectively, were predominant in the NTC2 as compared to the other samples. For this reason, we decided to remove these taxa from all samples of the study. Halomonadaceae and Flavomonadaceae are already known as reagent contaminant (Salter at al., 2014). https://bmcbiol.biomedcentral.com/articles/10.1186/s12915-014-0087-z. Weyrichh et al., 2018 ttps://www.biorxiv.org/content/biorxiv/early/2018/11/02/460212.full.pdf). A well-well contamination can be hypothesized for what concerns the other taxa, as the quantification is predominant in all the other samples and the other taxa are common skin resident bacteria (Minick et al., 2019 https://msystems.asm.org/content/msys/4/4/e00186-19.full.pdf).The taxonomy results of all samples including negative controls, without any taxa filtering, is available in Additional file 2.

```
#Filter for Flavobacteriaceae and Halomonadaceae (contaminants)
qiime taxa filter-table \
  --i-table tableV4-SILVA-noChlor.qza \
  --i-taxonomy newtaxonomySILVA-without-spacesmct.qza \
  --p-exclude Flavobacteriaceae,Halomonadaceae \
  --o-filtered-table table-no-halo-flavo.qza &
```

We rarefied the table at 4500 and at 28300 sequences. As we got the same results, we decided to include in the analysis more samples as possible

```
# Filter samples with a number of reads equal or higher than 4500
qiime feature-table filter-samples \
  --i-table table-no-halo-flavo.qza \
  --m-metadata-file ./4500/metadata4500.tsv \
  --o-filtered-table ./4500/id-filtered-4500.qza &

qiime feature-table summarize \
  --i-table ./4500/id-filtered4500.qza \
  --o-visualization ./4500/id-filtered-4500.qzv &

# Perform alpha and beta diversity
qiime diversity core-metrics-phylogenetic \
  --i-phylogeny rooted-tree.qza \
  --i-table ./4500/id-filtered-4500.qza \
  --p-sampling-depth 4500 \
  --m-metadata-file ./4500/metadata4500.tsv \
  --output-dir ./4500/core-metrics-results_MCT4500 &

# Observed species analysis
```

```
qiime metadata tabulate \
  --m-input-file ./4500/core-metrics-results_MCT4500/observed_otus_vector.qza \
  --o-visualization ./4500/core-metrics-results_MCT4500/observed_otus_vector.qzv &

# Shannon index analysis
qiime metadata tabulate \
  --m-input-file ./4500/core-metrics-results_MCT4500/shannon_vector.qza \
  --o-visualization ./4500/core-metrics-results_MCT4500/shannon_vector.qzv &

# Perform alpha-rarefaction
qiime diversity alpha-rarefaction \
  --i-table ./4500/id-filtered-4500.qza \
  --i-phylogeny rooted-tree.qza \
  --p-max-depth 4500 \
  --m-metadata-file ./4500/metadata4500.tsv \
  --o-visualization ./4500/alpha-rarefaction_mct4500.qzv &

# Extract the matrices from unweighted and weighted UniFrac
and bray curtis analysis
qiime tools export \
  ./4500/core-metrics-results_MCT4500/bray_curtis_distance_matrix.qza \
  --output-dir ./4500/core-metrics-results_MCT4500/
  exported-feature-table-bray_curtis &

## Taxonomy
# From absolute to relative abundance (for both phyla and families)
qiime taxa collapse \
  --i-table ./Taxonomy/tableV4-SILVA-noChlor.qza \
  --i-taxonomy taxonomySILVA-dadaV4.qza \
  --p-level 5 \ # Level 5 (Family level); Level 2 (Phylum level)
  --o-collapsed-table ./Taxonomy/taxonomy-L5.qza &

qiime feature-table relative-frequency \
  --i-table ./Taxonomy/taxonomy-L5.qza \
  --o-relative-frequency-table ./Taxonomy/taxonomy-L5-Relativa.qza &

qiime tools export \
  --input-path ./Taxonomy/taxonomy-L5-Relativa.qza \
  --output-path ./Taxonomy/exported-feature-table-relativa_L5 &

biom convert \
  -i ./Taxonomy/exported-feature-table-relativa_L5/feature-table.biom \
  -o ./Taxonomy/exported-feature-table-relativa_L5/
  Relativa-L5.txt --to-tsv --header-key taxonomy &
```

Statistic analysis

As already reported in the manuscript, XLSTAT software was used for the statistical analysis. The data distribution was tested by Shapiro test. T-, wilcoxon-signed and Mann-whitney tests were applied depending on the normality of each dataset distribution.

Box plot visualization in R

```
#Import the file with three columns: 'Group', 'Distance' and 'Type'.
The 'Group' column contains the status together with the tissue
(e.g. healthy - skin); the 'Distance' column contains the distance matrices;
the 'Type' column contains the method used together with the
tissue (e.g. unwehted UniFrac - Skin)

library(readxl)
BoxPlot_R_MCT <- read_excel("---/BoxPlot_R_MCT.xlsx",
                            sheet = "BoxPlot")
View(BoxPlot_R_MCT)
df= BoxPlot_R_MCT

# Load ggplot2 package
library(ggplot2)

# Order the column 'Type'
library(ggsignif)
df$Type <- factor(df$Type,
                  levels = c('unweighted_skin','weighted_skin',
                  'bray-curtis_skin','unweighted_biopsy',
                  'weighted_biopsy','bray-curtis_biopsy'))

# Perform the box plot
dp <- ggplot(data=df, aes(x=df$Group, y=df$Distance, fill=df$Group)) +
  geom_boxplot(aes(fill = df$Group))+ stat_summary(fun.y=mean, geom = "point")+
  labs(title="",x="", y = "")+
  facet_grid( ~ df$Type, scales="free_x", space = "free")+
  theme(panel.spacing = unit(.05, "lines"),
        panel.border = element_rect(color = "black", fill = NA, size = 3),
        strip.background = element_rect(color = "black", size = 3))

dp + scale_fill_brewer(palette="Pastel1") + theme_minimal()
+ theme(axis.text.x = element_text(angle= 90))

# Save the figure
ggsave("Beta-diversity_MCT_Dog1200dpi.png",height=5,width=8,dpi=1200)
```

Bar plot at phylum and family level in R

```
# Import the file with the following columns: 'Factor A'
(with the rows numbers), 'Factor B' (with the status:
healthy or tumor), 'Animal' (with the dog ID), 'Site'
(with the status together with the tissue - e.g. tumor skin),
Phyla or families columns
# All taxa considered for the bar plot have a relative
abundance equal or more than 2%.

## For Phyla
library(readxl)
Images_Taxo <- read_excel("---/Images_Taxo.xlsx",
                          sheet = "Phylum_Paird_+Bio >=2%")
View(Images_Taxo)
```

```
df=Images_Taxo_group

df$Actinobacteria <- as.vector(as.list(df$Actinobacteria))
df$Bacteroidetes <- as.vector(as.list(df$Bacteroidetes))
df$Firmicutes <- as.vector(as.list(df$Firmicutes))
df$Fusobacteria <- as.vector(as.list(df$Fusobacteria))
df$Proteobacteria <- as.vector(as.list(df$Proteobacteria))
df$Tenericutes <- as.vector(as.list(df$Tenericutes))

library(data.table)
setDT(df)
melt.data <- melt(df,id=c("Factor_A", "Factor_B", "Animal", "Site"))

melt.data

library(ggplot2)
library(reshape2)
library(dplyr)

melt.data$value <- as.numeric(melt.data$value)
melt.data$value

melt.data$Animal = as.character(melt.data$Animal)
melt.data$Site = as.character(melt.data$Site)

melt.data <- with(melt.data, melt.data[order
(melt.data$Animal, melt.data$value, melt.data$Site),])

# order characters
library(ggsignif)
melt.data$Site <- factor(melt.data$Site,levels
= c('Healthy Skin','Tumor Skin','Tumor Dermis'))

# Perform the bar plot
p<- ggplot(data=melt.data, aes(x=melt.data$Site, melt.data$value, fill=melt.data$variable)) +
  geom_bar(stat="identity")

#PALETTE6COLORS_mixedcolors
p + scale_fill_manual(values=c("#99CC00","#660033",
"#99CC99","#FFCCFF","#CC9966","#FFFF66"))+
  theme_light() + theme(axis.text.x =
  element_text(angle= 50, hjust = 1, size = 20,
  color = "black"),
                      axis.text.y.left =
                      element_text
                      (color = "black",  size = 20),
                      legend.text =
                      element_text(colour = "black",
                      size = 20),
                      strip.background =
                      element_rect(fill = "white"),
                      strip.text = element_text(colour =
                      "black",size = 20),
                      panel.grid.major = element_blank(),
```

```
                        panel.grid.minor = element_blank(),
                        panel.border =
                        element_rect(colour = "white"))


ggsave("NEW_group_Phylum_Taxonomy_MCT_Dog1200dpi.png",height=10,width=8,dpi=1200)




##For Families
library(readxl)
Images_Taxo <- read_excel("---/Images_Taxo.xlsx",
                          sheet = "Family_Paird_+Bio >=2%")
View(Images_Taxo)

df=Images_Taxo_group

df$`Clostridiaceae 1` <- as.vector(as.list(df$`Clostridiaceae 1`))
df$Corynebacteriaceae <- as.vector(as.list(df$Corynebacteriaceae))
df$Cytophagaceae <- as.vector(as.list(df$Cytophagaceae))
df$Fusobacteriaceae <- as.vector(as.list(df$Fusobacteriaceae))
df$Lachnospiraceae <- as.vector(as.list(df$Lachnospiraceae))
df$Micrococcaceae <- as.vector(as.list(df$Micrococcaceae))
df$Moraxellaceae <- as.vector(as.list(df$Moraxellaceae))
df$Mycoplasmataceae <- as.vector(as.list(df$Mycoplasmataceae))
df$Nocardioidaceae <- as.vector(as.list(df$Nocardioidaceae))
df$Nocardiaceae <- as.vector(as.list(df$Nocardiaceae))
df$Pasteurellaceae <- as.vector(as.list(df$Pasteurellaceae))
df$Peptostreptococcaceae <- as.vector(as.list(df$Peptostreptococcaceae))
df$Planococcaceae <- as.vector(as.list(df$Planococcaceae))
df$Porphyromonadaceae <- as.vector(as.list(df$Porphyromonadaceae))
df$Propionibacteriaceae <- as.vector(as.list(df$Propionibacteriaceae))
df$Ruminococcaceae <- as.vector(as.list(df$Ruminococcaceae))
df$Shewanellaceae <- as.vector(as.list(df$Shewanellaceae))
df$Staphylococcaceae <- as.vector(as.list(df$Staphylococcaceae))
df$Streptococcaceae <- as.vector(as.list(df$Streptococcaceae))
df$Aerococcaceae <- as.vector(as.list(df$Aerococcaceae))
df$Erysipelotrichaceae <- as.vector(as.list(df$Erysipelotrichaceae))
df$Rhodobacteraceae <- as.vector(as.list(df$Rhodobacteraceae))

library(data.table)
setDT(df)
melt.data <- melt(df,id=c("Factor_A", "Factor_B", "Site"))

melt.data

library(ggplot2)
library(reshape2)
library(dplyr)

melt.data$value <- as.numeric(melt.data$value)
melt.data$value
```

```
melt.data$Site = as.character(melt.data$Site)

melt.data <- with(melt.data, melt.data[order(melt.data$value, melt.data$Site),])

##order characters

library(ggsignif)
melt.data$Site <- factor(melt.data$Site,levels = c('Healthy Skin','Tumor Skin','Tumor Dermis'))

p<- ggplot(data=melt.data, aes(x=melt.data$Site, melt.data$value, fill=melt.data$variable)) +
  geom_bar(stat="identity")


##PALETTE22COLORS_mixedcolors
p + scale_fill_manual(values=c("#99CC00","#660033",
"#006633","#CC0033","#66FFCC","#CC6666","#99CC99",
"#FFCCFF","#CC9900","#990000","#009966","#660066",
"#003333","#CC9966","#FF9900","#333366","#FFFF66",
"#FF9966","#6699CC","#CC3366","#993300","#FF66FF")) +
  theme_light() + theme(axis.text.x =
  element_text(angle= 50, hjust = 1, size = 20,
  color = "black"),
                      axis.text.y.left =
                      element_text(color =
                      "black",  size = 20),
                      legend.text =
                      element_text(colour =
                      "black", size = 20),
                      strip.background =
                      element_rect(fill = "white"),
                      strip.text = element_text(colour =
                      "black",size = 20),
                      panel.grid.major = element_blank(),
                      panel.grid.minor = element_blank(),
                      panel.border =
                      element_rect(colour = "white"))+
                      guides(fill=guide_legend(nrow = 22))


ggsave("New_group_Family_Taxonomy_MCT_Dog1200dpi.png",
height=10,width=9,dpi=1200)
```

MDS and PCA analysis in R

```
# This script performs multidimensional scaling and
# Pricipal Component analysis on the ASV matrix, filtering
# out non-variable Phyla/Families.
#
# It generates the MDS for both individuals and Phyla/Families,
# saving the eigenvalues for every phylum/family.
# It also run the PCA on the same dataset and save the contributions
# Of every variable to the first component.
#
```

```
# All the results are saved into a subfolder with name specified
# into the variable outname. It saves MDS plots, PCA plots and
# eigenvalues/contributions as pdf, and tables produced by the analyses
# in an excel workbook, with one worksheet per table.
#


# Setting options
options(digits = 22, stringsAsFactors = F)

# Importing packages
if (!require(FactoMineR)) install.packages("FactoMineR")
if (!require(factoextra)) install.packages("factoextra")
if (!require(ggplot2)) install.packages("ggplot2")
if (!require(ggrepel)) install.packages("ggrepel")
if (!require(openxlsx)) install.packages('openxlsx')
library(FactoMineR)
library(factoextra)
library(ggplot2)
library(ggrepel)
library(openxlsx)

## Function to add worksheets to an excel file
addSheet = function(data, name){
  addWorksheet(wb, name)
  writeData(wb, name, data, rowNames = T)
}


# Import dataset
# If importing from windows, replace pipe("pbpaste")
# with clipboard
# Change the outname (for folder and prefix in output files)
# Change target column with Phylum/Family
# Threshold is the ratio of non-zero values in every phylum/family
wb <- createWorkbook()
mtrx = read.table(pipe("pbpaste"), h=T, sep = "\t")
outname = "PhylumSkin"
tgtCol = "Phylum"
threshold  = .5

# Generate output folder and enter it
dir.create(outname)
setwd(outname)

# Start analysis
rownames(mtrx) = mtrx[,tgtCol]  # Give row names
mtrx[,tgtCol] = NULL  # Remove the column with names
mtrx = mtrx[rowSums(mtrx > 0) / dim(mtrx)[2] > threshold,]  # Filter out non-variable phyla/famil
mtrx = mtrx[rownames(mtrx) != "__", ] # Remove __ family/phyla
stds = apply(mtrx, 1, sd)  # Calculate StDs for every
mtrx = mtrx[rownames(mtrx)[stds!=0],]  # Keep columns with StD > 0
addSheet(mtrx, "FilteredMatrix")
```

```
# MDS analysis
tmtrx = t(mtrx) # Transpose matrix

# Create sample ids. If the sample coding change, this MUST changed too
samples = data.frame(ID=rownames(tmtrx)) %>% separate(ID, c("SampleID", "Animal", "Tissue", "Statu
samples$ID = paste(samples$SampleID, samples$Animal, samples$Tissue, samples$Status, sep = "_") #

# Calculate distances and MDS for individuals
ind_dists = dist(x = tmtrx, method = "manhattan")
post_mds_id<-cmdscale(d=ind_dists, k = 5, eig = T)
to_plot_inds = cbind(samples, post_mds_id$points)
addSheet(to_plot_inds, "MDScomponents")

# Create MDS plot
pdf(paste("MDS_plot_", outname, ".pdf", sep = ""))
p = to_plot_inds %>% ggplot(aes(x = `1`, y = `2`, colour = Animal, label = ID)) +
  geom_point() +
  geom_label_repel()
p
dev.off()

# Get eigenvalues for phyla/families
phylas = data.frame(ST=row.names(mtrx))
sp_dists = dist(x = mtrx, method = "manhattan")
post_mds_sp<-cmdscale(d=sp_dists, k = 5, eig = T)
to_plot_sp = cbind(phylas, post_mds_sp$points)
addSheet(to_plot_inds, "MDScomponents_OTU")

# Make MDS plot for variables
pdf(paste("MDS_plot_", outname, ".pdf", sep = ""))
p = to_plot_sp %>% ggplot(aes(x = `1`, y = `2`, label = ST)) +
  geom_point() +
  geom_label_repel()
p
dev.off()

# Define eigenvalues for variables in MDS plot
eigv = data.frame(ST = phylas$ST, Eigenv = post_mds_sp$eig)
eigv = eigv[order(eigv$Eigenv, decreasing = T),]
eigv$ST <- factor(eigv$ST, levels=as.character(eigv$ST))
addSheet(eigv, "MDS_eigenvals")

# Save plot
pdf(paste("MDS_plot_Eigenv_", outname, ".pdf", sep = ""))
p = eigv %>% ggplot(aes(x = ST, y = Eigenv)) +
  geom_bar(stat = "identity", fill = "blue") +
  theme(axis.text.x=element_text(angle=90,hjust=1,vjust=0.5))
p
dev.off()

### Run PCA on the dataset and save results
re.pca = PCA(tmtrx, graph = T)
to_plot_pca = cbind(samples, re.pca$ind$coord)
addSheet(to_plot_pca, "PCAcomponents")
```

```
# Plot PCA
pdf(paste("PCA_plot_", outname, ".pdf", sep = ""))
p = to_plot_pca %>% ggplot(aes(x = `Dim.1`, y = `Dim.2`, label = ID, colour = Status)) +
  geom_point() +
  geom_label_repel()
p
dev.off()

# Save contributions of Phyla/Families to data
contrib = as.data.frame(re.pca$var$contrib)
contrib = contrib[order(contrib[,1], decreasing = TRUE),]
contrib$ST = rownames(contrib)
contrib$ST <- factor(contrib$ST, levels=as.character(contrib$ST))
addSheet(contrib, "PCcontributions")

# Save contribution plot
pdf(paste("PCA_plot_contrib_", outname, ".pdf", sep = ""))
p = contrib %>% ggplot(aes(x = ST, y = `Dim.1`)) +
  geom_bar(stat = "identity", fill = "blue") +
  theme(axis.text.x=element_text(angle=90,hjust=1,vjust=0.5))
p
dev.off()

# Save excel workbook
saveWorkbook(wb, file = paste(outname,"_results.xlsx"), overwrite = TRUE)
rm(wb)

# Go back in folder
setwd("../")
```
