# Supplementary material for: Characterization of skin surface and dermal microbiota in dogs with mast cell tumor
Source: Sci Rep. 2020 Jul 28;10:12634. doi: 10.1038/s41598-020-69572-0 (PMC7387470; doi:10.1038/s41598-020-69572-0)
Supplement: Supplementary file 5 — Supplementary file5 (PDF 552 kb) [file 41598_2020_69572_MOESM5_ESM.pdf]

Supplementary Table S3.

Characterization of skin surface and dermal microbiota in dogs with mast cell tumor

Valentina Zamarian[1#], Carlotta Catozzi[1#], Anna Cuscó[2], Damiano Stefanello[1], Roberta Ferrari[1], Fabrizio Ceciliani[1], Olga Francino[3], Armand Sánchez[3], Valeria Grieco[1], Davide Zani[1], Andrea Talenti[4], Paola Crepaldi[5], Cristina Lecchi[1*]

(1) Dipartimento di Medicina Veterinaria, Università degli Studi di Milano, Milano, Italy
(2) Vetgenomics. Ed Eureka. PRUAB. Campus UAB, Barcelona, Spain
(3) Molecular Genetics Veterinary Service (SVGM), Veterinary School, Universitat Autònoma de Barcelona, Barcelona, Spain
(4) The Roslin Institute, University of Edinburgh, Easter Bush Campus, Midlothian, EH25 9RG, United Kingdom
(5) Department of Agricultural and Environment Science, Università degli Studi di Milano, Milano, Italy.

#Equal contribution

Supplementary table S3. The number of observed OTUs detected in tumor and heathy skin surface and in tumor dermis samples.

Sequencing sample ID	*Patient*	*Status*	*Observed OTU*
M003	Dog 2	Tumor skin surface	198
M004	Dog 2	Healthy skin controlateral	183
M007	Dog 3	Tumor skin surface	95
M008	Dog 3	Healthy skin controlateral	599
M009	Dog 4	Tumor skin surface	367
M010	Dog 4	Healthy skin controlateral	901
M017	Dog 5	Tumor skin surface	191
M018	Dog 5	Healthy skin controlateral	930
M020	Dog 6	Healthy skin controlateral	1029
M021	Dog 7	Tumor skin surface	904
M022	Dog 7	Healthy skin controlateral	383
M025	Dog 9	Tumor skin surface	178
M027	Dog 10	Tumor skin surface	155
M030	Dog 11	Healthy skin controlateral	102
M031	Dog 1	Tumor dermis biopsy	96
M032	Dog 2	Tumor dermis biopsy	95
M035	Dog 4	Tumor dermis biopsy	108
M043	Dog 10	Tumor dermis biopsy	218
M045	Dog 11	Tumor dermis biopsy	101
