# Supplementary material for: Characterization of skin surface and dermal microbiota in dogs with mast cell tumor
Source: Sci Rep. 2020 Jul 28;10:12634. doi: 10.1038/s41598-020-69572-0 (PMC7387470; doi:10.1038/s41598-020-69572-0)
Supplement: Supplementary file 7 — Supplementary file7 (PDF 576 kb) [file 41598_2020_69572_MOESM7_ESM.pdf]

Supplementary Table S5.

Characterization of skin surface and dermal microbiota in dogs with mast cell tumor

Valentina Zamarian[1#], Carlotta Catozzi[1#], Anna Cuscó[2], Damiano Stefanello[1], Roberta Ferrari[1], Fabrizio Ceciliani[1], Olga Francino[3], Armand Sánchez[3], Valeria Grieco[1], Davide Zani[1], Andrea Talenti[4], Paola Crepaldi[5], Cristina Lecchi[1*]

[(1)] Dipartimento di Medicina Veterinaria, Università degli Studi di Milano, Milano, Italy
[(2)] Vetgenomics. Ed Eureka. PRUAB. Campus UAB, Barcelona, Spain
[(3)] Molecular Genetics Veterinary Service (SVGM), Veterinary School, Universitat Autònoma de Barcelona, Barcelona, Spain
[(4)] The Roslin Institute, University of Edinburgh, Easter Bush Campus, Midlothian, EH25 9RG, United Kingdom
[(5)] Department of Agricultural and Environment Science, Università degli Studi di Milano, Milano, Italy.
#Equal contribution

Supplementary Table S5. Data of dogs enrolled in the study. F= female; M= male; += MCT- affected dog; -= healthy dog; N/A= not applicable.

Animal	*Sex*	*Age (years)*	*Breed*	*Tumor*	*Onset site*	*Patnaik*	*Kiupel*
Dog 1	F	10	Cocker	+	Cutaneous	II	Low
Dog 2	M	10	Labrador	+	Cutaneous	II	Low
Dog 3	M	12	Mixed breed	+	Cutaneous	II	Low
Dog 4	F	4	Swiss Mountain dog	+	Cutaneous	II	Low
Dog 5	M	7	Weimaraner	+	Cutaneous	II	Low
Dog 6	F	6	Labrador	+	Cutaneous	II	Low
Dog 6	M	1	Labrador	+	Cutaneous	I	Low
Dog 8	F	9	Setter	+	Subcutaneous	II	N/A
Dog 9	M	6	Mixed breed	+	Subcutaneous	II	N/A
Dog 10	M	10	Shar-pei	+	Subcutaneous	II	N/A
Dog 11	F	14	Mixed breed	+	Subcutaneous	II	N/A
Dog 12	F	3	Mixed breed	-			
Dog 13	F	2	Mixed breed	-			
Dog 14	M	2	Mixed breed	-			
